# Synthesis of Oxazinanones: Intramolecular Cyclization of Amino Acid-Derived Diazoketones via Silica-Supported HClO_4_ Catalysis

**DOI:** 10.3389/fchem.2019.00062

**Published:** 2019-02-08

**Authors:** Rafael D. C. Gallo, Orlando C. Campovilla Jr., Anees Ahmad, Antonio C. B. Burtoloso

**Affiliations:** Instituto de Química de São Carlos, Universidade de São Paulo, São Carlos, Brazil

**Keywords:** heterocycles, oxazinanones, intramolecular cyclization, Brønsted acid, diazo carbonyls

## Abstract

A Brønsted acid catalyzed intramolecular cyclization of *N*-Cbz-protected diazoketones, derived from α-amino acids, is described. The reaction proceeds under metal-free conditions and is promoted by ecofriendly silica-supported HClO_4_ as the catalyst and methanol as the solvent. This transformation enables the short synthesis of various 1,3-oxazinane-2,5-diones under mild reaction conditions and in good yields (up to 90%). The set-up is very simple; by just mixing all reagents together with no work-up necessary before purification, this protocol takes a greener approach.

## Introduction

**Figure 1:**
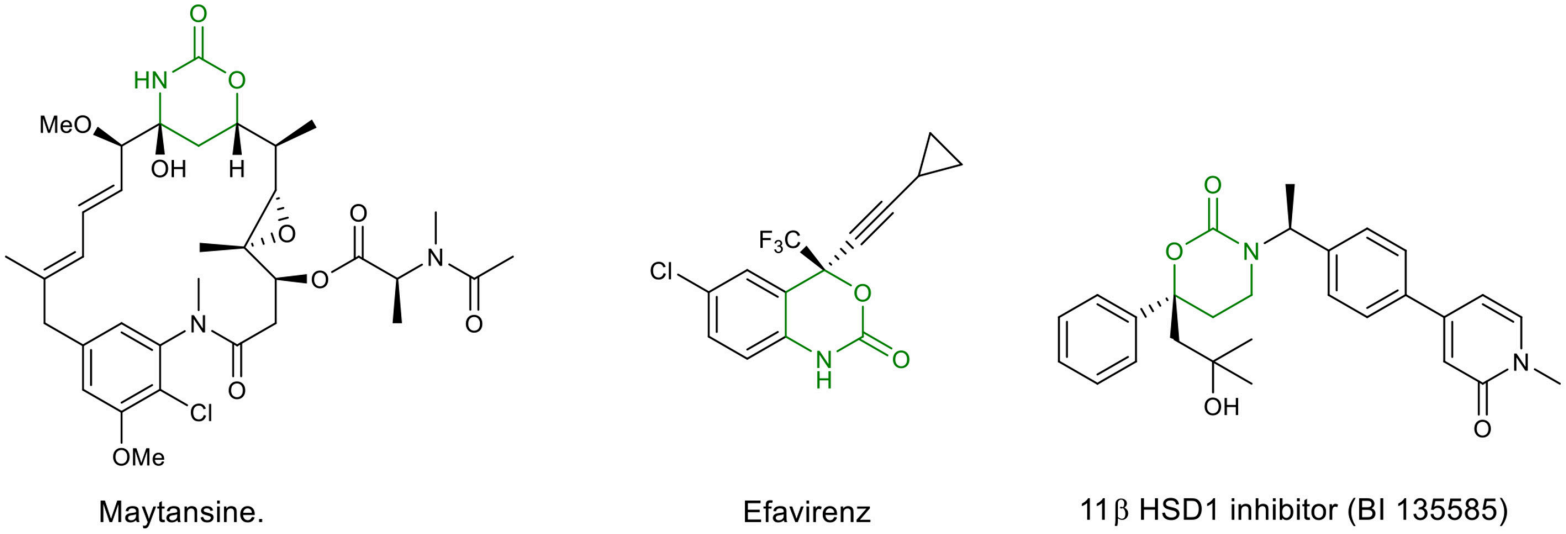
Bioactive molecules bearing an oxazinanone moiety.

Oxazinanones (six-membered cyclic urethanes) are an important class of heterocycles, which have been found to be key structural units in bioactive natural products and pharmaceutically important molecules. Some important examples are the anti-HIV drug Efavirenz (Staszewski et al., 1999) and the potent anticancer agent Maytansine (Rao et al., 1979) and its synthetic derivatives Maytansinoid (Blanc et al., 2011) and Ansamitocin P3 (Taft et al., 2012). Other significant biological activities described for this class of compounds are: antibacterial (Zanatta et al., 2006; Wang, 2008), anti-influenza (Kuznetsov et al., 2017), anti-inflammatory (Ullrich et al., 2004), antidiabetes [11β HSD1 inhibitor (BI 135585)] (Zhuang et al., 2017), antithrombotic (Jin and Confalone, 2000), antialzheimer (Fuchs et al., 2007), and enzyme inhibiting [(Latli et al., 2017); Figure 1]. Furthermore, they are extensively used as valuable synthetic intermediates in fine chemicals (Woodward et al., 1981; Wang et al., 1982; Hilborn et al., 2001; Takahata et al., 2002; Wang and Tunge, 2006), cosmetics (Zofchak, 2003), and pesticides (Hino et al., 2008). They have also showed wide applications as ligands, auxiliaries and as phase transfer catalysts in organic synthesis (Davies et al., 2006; Lait et al., 2007).

**Figure 2:**
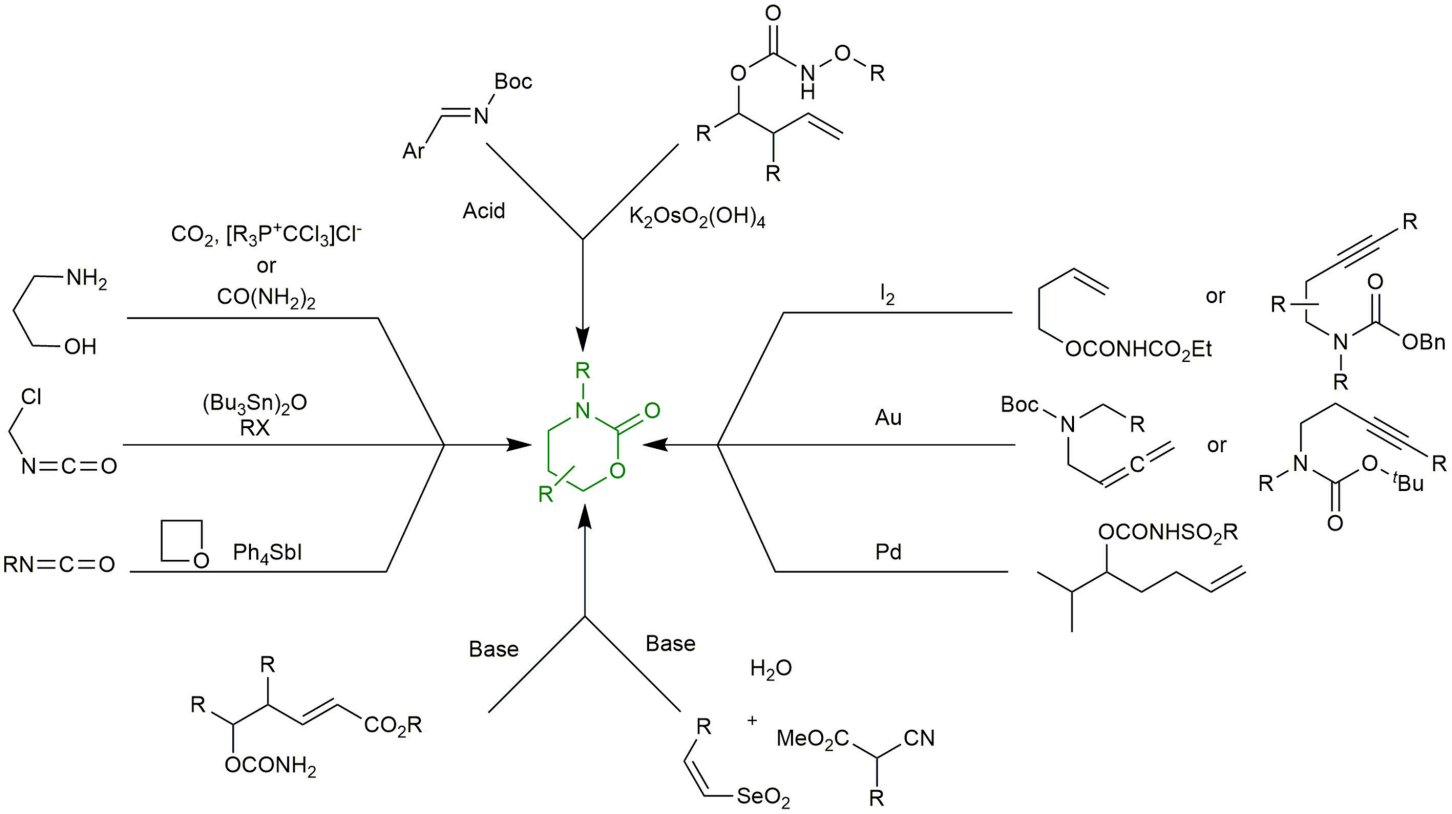
Synthetic methodologies for the preparation of oxazinanone moiety.

Thus, it is therefore not surprising that various synthetic methods for the construction of 1,3-oxazinan-2-one rings have been reported in literature. Among many current methodologies, reactions of CO_2_ (Kubota et al., 1993) or Urea (Bhanage et al., 2004) with amino alcohols, cycloaddition of isocyanates to oxetanes (Fujiwara et al., 1989), coupling of the adducts from the reaction between (Bu_3_Sn)_2_O and haloalkyl isocyanate with alkyl halides (Shibata et al., 1989), iodine-mediated (Fujita et al., 1997; Quinodoz et al., 2017), gold-catalyzed (Robles-Machín et al., 2006; Alcaide et al., 2013), Pd/sulfoxide-catalyzed C-H amination (Rice and White, 2009), intramolecular Michael addition reactions (Hirama et al., 1985) of appropriately functionalized allylic/homoallylic/homopropargyl/allenic carbamates, tethered aminohydroxylation (Donohoe et al., 2007), Brønsted base catalyzed Michael addition of α-isocyanoacetates to phenyl vinyl selenones (followed by domino oxidative cyclization) (Buyck et al., 2014) and Brønsted acid catalyzed elimination-cycloaddition reaction of Boc-imines (Uddin et al., 2011) are the most interesting ones (Figure 2).

**Figure 3:**
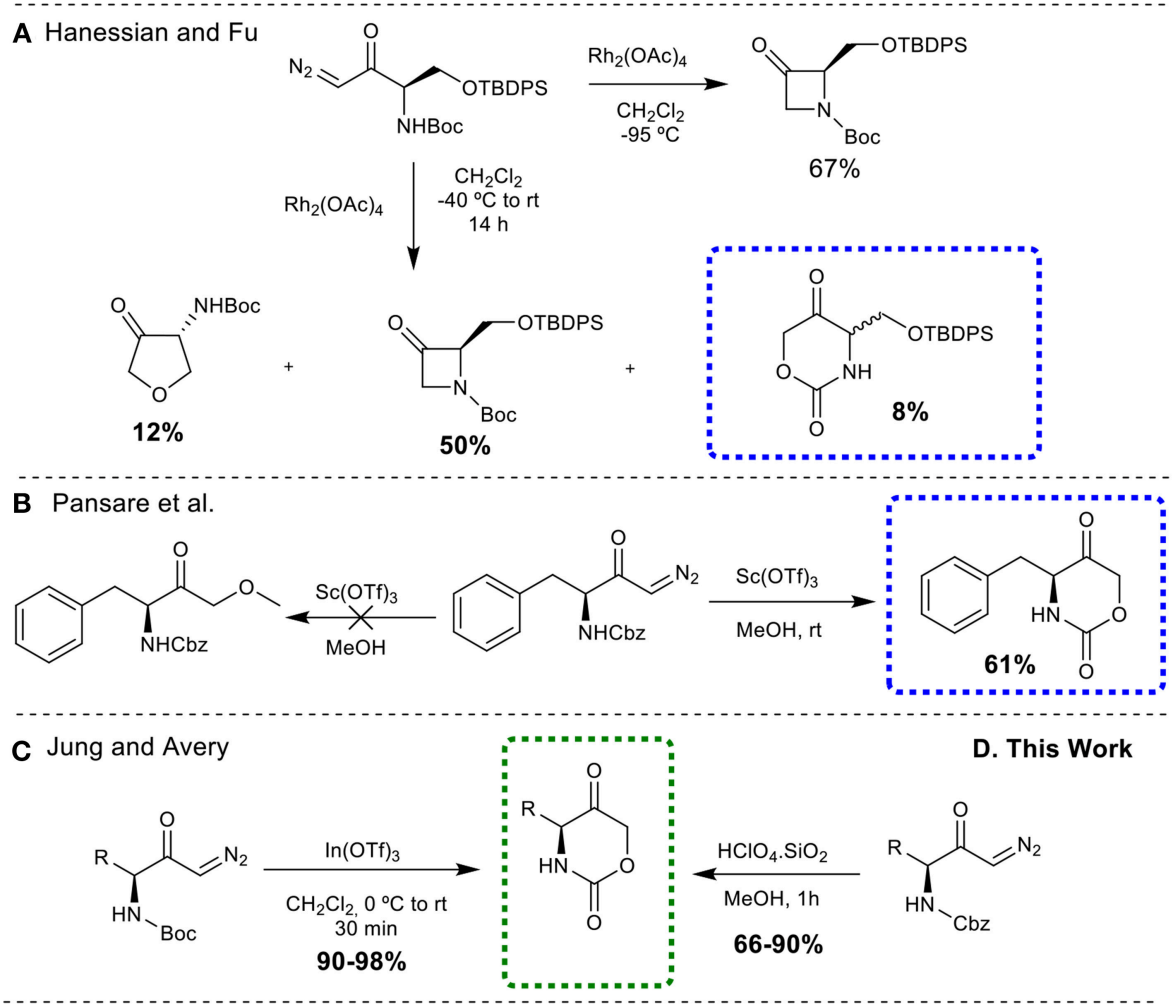
Synthesis of 1,3-oxazinane-2,5-diones from diazo carbonyl moieties. **(A)** Through rhodium catalyzed N-H insertion reaction; **(B)** through scandium triflate (Sc(OTf)_3_) catalyzed insertion reaction; **(C)** through an indium triflate [In(OTf)_3_] catalyzed intramolecular cyclization reaction; **(D)** through silica-supported HClO_4_ catalyzed intramolecular cyclization reaction.

All the methodologies described above are dedicated to 1,3-oxazinan-2-one skeletons, whereas only a few are reported for the preparation of 1,3-oxazinane-2,5-dione rings. Hanessian and Fu (2001) described the synthesis of this class of compound as a by-product, during a rhodium catalyzed N-H insertion reaction of a diazoketone (synthesis of 3-azetidinones) (Figure 3A). Pansare et al. (1999) treated a diazoketone derived from *N*-Cbz-phenylalanine with scandium triflate (Sc(OTf)_3_) as the catalyst in methanol, to obtain the oxazinanedione moiety (Figure 3B). Similarly, Jung and Avery (2006) successfully demonstrated the synthesis of cyclic urethanes from Boc-protected diazocarbonyl substrates through an indium triflate [In(OTf)_3_] catalyzed intramolecular cyclization reaction (Figure 3C).

Despite the fact that numerous modern, scalable and greener methods to obtain diazo carbonyl compounds were described over the past few years (Maas, 2009; Burtoloso et al., 2018), the development of greener, metal free, cheap and easily available catalysts for the efficient synthesis of cyclic urethanes is still highly desirable. With these demands in mind, efforts have been made to use Brønsted acid catalyst as a potential substitute to perform the desired transformation. It is also important to mention here that our group has developed an O-H insertion reaction into diazo carbonyl compounds employing Bronsted acid catalyst (Gallo and Burtoloso, 2018). Herein, we report the operationally simple and greener synthesis of 1,3-oxazinane-2,5-diones via silica-supported HClO_4_ catalyzed cyclization of *N*-Cbz-protected diazoketones, offering an interesting alternative to the existing synthetic methods (Figure 3D). Although a single example for a *N*-Boc-protected diazoketone was described by Jung and Avery with HClO_4_, the conditions employed (solution in CH_2_Cl_2_) and the use of Boc protecting group makes this method less interesting when compared to the present protocol.

## Results and Discussion

We initiated our screening by selecting phenylalanine-derived *N*-Cbz-protected diazoketone **1** as the model substrate and investigated its behavior under different reaction conditions (Table 1). Based on our previous work (Gallo and Burtoloso, 2018), compound **1** was simply mixed with 10 mol% of H_2_SO_4_ (pKa = −3.0) as the BrØnsted acid in benzyl alcohol (BnOH) as the solvent for 24 h at room temperature. To our delight, we isolated the intramolecular cyclization product, oxazinanone **2**, in 12% yield instead of getting O-H insertion product (entry 1). A slight improvement in the yield was observed while using stronger acid HClO_4_ (pKa = −10) as the catalyst under similar reaction conditions (entry 2). In order to minimize side product formation, as well as ease in acid handling, H_2_SO_4_ was immobilized on silica gel (230–400 mesh) (Chakraborti and Gulhane, 2003; Chakraborti and Chankeshwara, 2006; Rudrawar et al., 2006). This manipulation proved to be useful, providing target molecule **2** with 35% yield (entry 3). Encouraged by this outcome, we used silica-supported HClO_4_ which led to a further increase in the yield of the reaction (entry 4). Significant increase in the yield (up to 62%) was noticed when EtOH was employed as a reaction medium (entry 5). Using MeOH as the solvent improved the reaction efficiency and the desired product **2** was isolated in 71% yield in shorter reaction times (12 h) (entry 6). Poor yield or no product formation was observed in the presence of non-nucleophilic solvents such as DCE, THF, and toluene (entries 7–9). Increasing catalyst loading to 20 and 30 mol % provided product **2** in 75 and 83% yield, respectively (entries 10 and 11). Further increasing the catalyst loading (40 mol %) did not affect the reaction yield (entry 12). Under similar conditions of entry 11, comparable yield (81%) of compound **2** was obtained when the reaction was carried-out during 1 h. Thus, conditions in entry 13 were chosen as the optimal to explore the scope of the reaction.

**Table 1:** Optimization conditions of Bronsted acid cyclization of diazo carbonyl **1**.

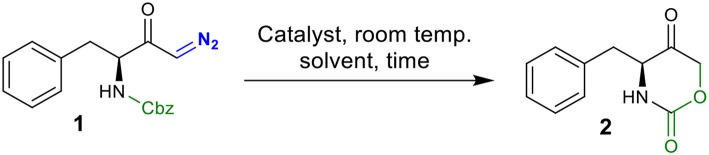					
**Entry**	**Catalyst**	**(mol%)**	**Solvent**	**Time (h)**	**Yield (%)**^**a**^
1	H_2_SO_4_	10	BnOH	24	12
2	HClO_4_	10	BnOH	24	27
3	H_2_SO_4_-SiO_2_	10	BnOH	24	35
4	HClO_4_-SiO_2_	10	BnOH	24	44
5	HClO_4_-SiO_2_	10	EtOH	24	62
6	HClO_4_-SiO_2_	10	MeOH	12	71
7	HClO_4_-SiO_2_	10	DCE	12	13
8	HClO_4_-SiO_2_	10	THF	12	0
9	HClO_4_-SiO_2_	10	Toluene	12	0
10	HClO_4_-SiO_2_	20	MeOH	12	75
11	HClO_4_-SiO_2_	30	MeOH	12	83
12	HClO_4_-SiO_2_	40	MeOH	12	82
13	HClO_4_-SiO_2_	30	MeOH	1	81

a *Isolated yield*.

**Scheme 1:**
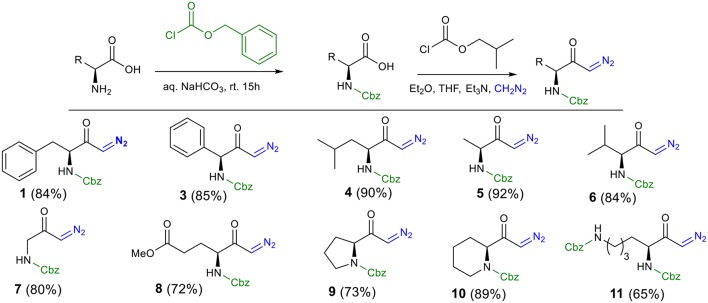
Preparation of *N*-Cbz-protected diazo carbonyls compounds **1** and **3**–**11**.

To explore the scope and generality of the reaction, we prepared several *N*-Cbz-protected diazoketones (**1** and **3–11**) with different substituents (Scheme 1, for detail procedure see Supplementary Material for the synthesis of diazoketones). In our approach, diazoketones **1** and **3**–**11** were accessed in excellent yields by protection of the respective amino acids with benzyl chloroformate in aqueous NaHCO_3_, followed by reaction with isobutyl chloroformate (to activate the carboxylic acid as a mixed anhydride) and freshly prepared diazomethane.

**Scheme 2:**
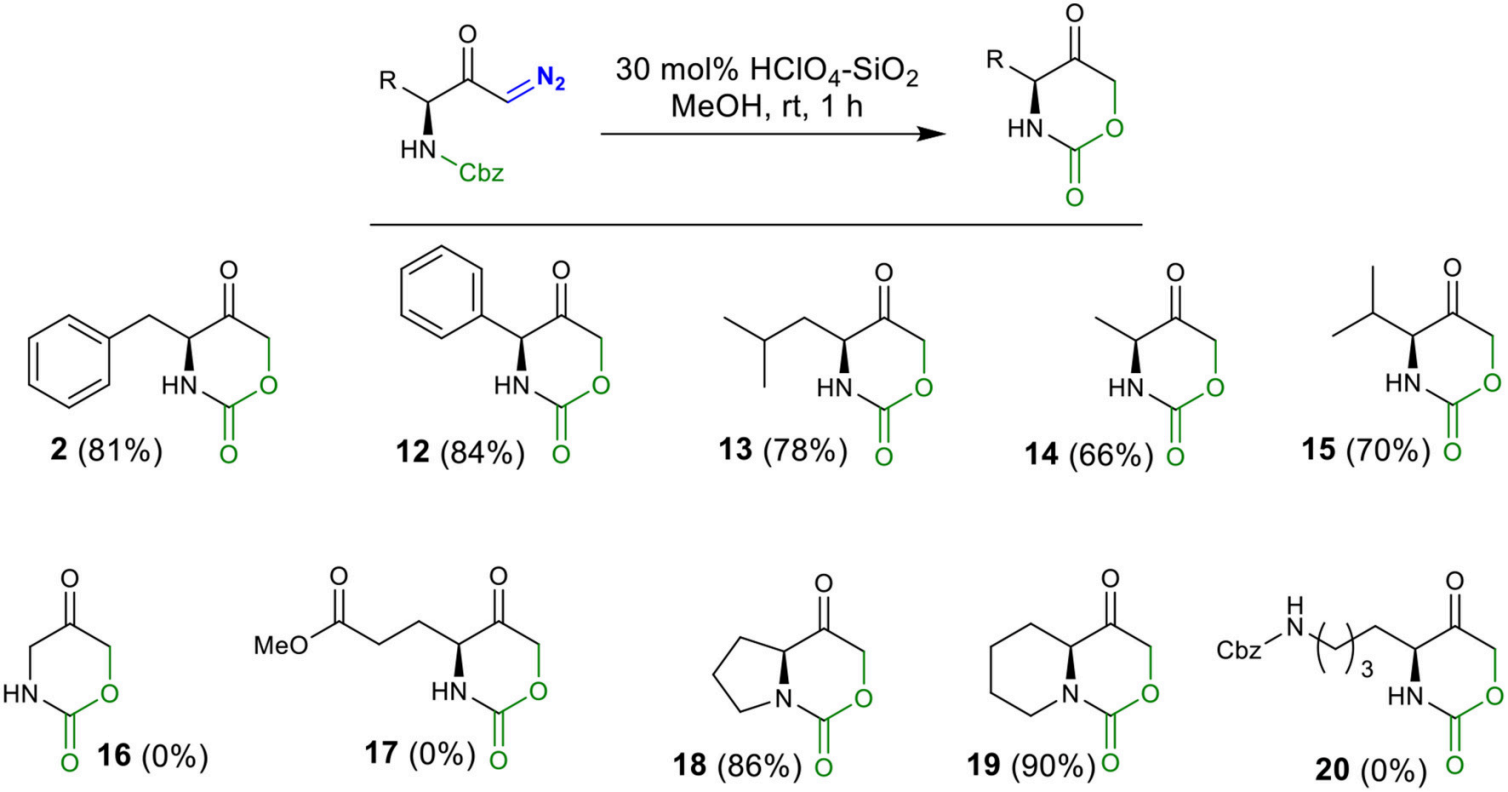
Synthesis of oxazinanones from diazo carbonyls via silica-supported HClO_4_ catalysis.

Employing the conditions from entry 13 (Table 1), the substrate scope was investigated (Scheme 2). The HClO_4_-SiO_2_ catalyst smoothly converted 2-phenylglycine derived diazo carbonyl **3** into cyclic urethane **12** in 84% yield. Similarly, for leucine-, alanine-, and valine-derived substrates **3**–**6**, the corresponding oxazinanones **13**–**15** were obtained in good yields. Surprisingly, no product formation was observed with glycine-derived diazo compound **7** under the standard reaction conditions (complex mixture) and this result is under investigation for a better understanding. Diazoketone **8**, possessing terminal ester functionality, also render no product. In the case of bicyclic oxazinanones **18** and **19**, derived from **9** and **10**, high yields were obtained. Finally, diazoketone **11**, with terminal Cbz-protected amine chain, did not provide the desired product.

Although merely speculative (studies are being carried-out), the two cbz groups in compound **11** can compete against each other for attack in the protonated diazo carbon (in an inter- or intramolecular fashion). In diazoketone **8**, the ester functionality can also compete with the cbz group during the insertion in the protonated diazo carbon. Moreover, the formation of the enol ether from **8** in acidic medium can furnish by-products through competing reactions.

**Figure 4:**
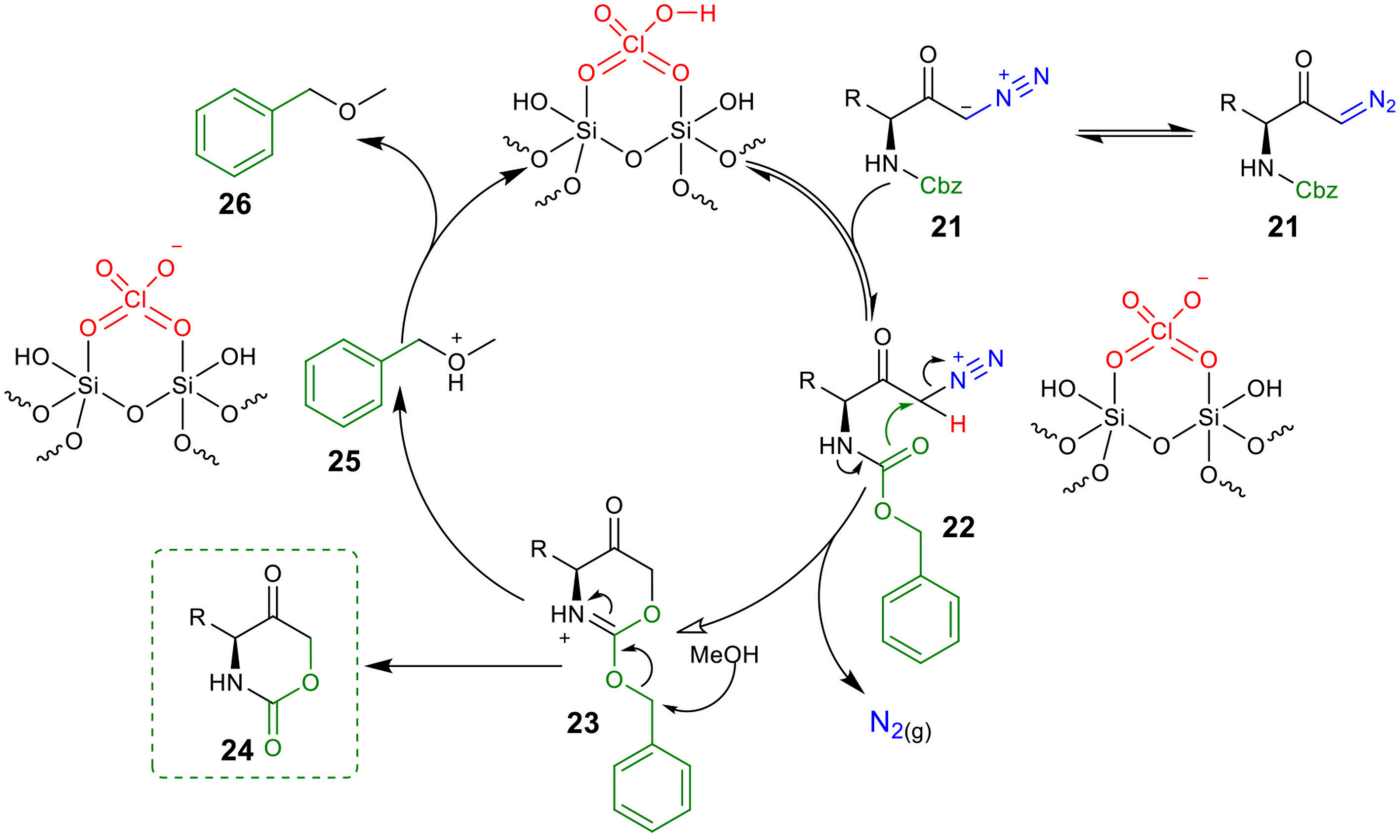
A proposed mechanism for the synthesis of oxazinanones from diazo carbonyls through silica-supported HClO_4_ catalysis.

Based on the above experimental results, a proposed mechanism to rationalize the formation of the 1,3-oxazinano-2,5-diones skeleton is shown in Figure 4. Protonation of diazo compound **21** by the Brønsted acid generates diazonium intermediate **22**. Next, the intramolecular nucleophilic attack from the Cbz carboxyl group at C1 releases molecular nitrogen and furnishes ammonium intermediated **23**. Finally, intermediate **23** is converted into the desired oxazinanone **24** after the nucleophilic attack of MeOH to the benzyl group. Hydrogen abstraction from **25** by the conjugate base of the catalyst regenerates the catalyst and provides (methoxymethyl)benzene **26** (detected by ^1^H NMR) as a byproduct.

In conclusion, we have disclosed a direct cyclization of amino acid-derived diazoketones via a silica-supported HClO_4_ catalysis, offering a practical and efficient route for the construction of several 1,3-oxazinane-2,5-diones in 66–90% yield. This protocol features a metal free, inexpensive, stable, and easy to handle catalyst, simple reaction set-up, short reaction time, non-chlorinated solvent and broad substrate scope.

## Author Contributions

All authors listed have made a substantial, direct and intellectual contribution to the work, and approved it for publication.

### Conflict of Interest Statement

The authors declare that the research was conducted in the absence of any commercial or financial relationships that could be construed as a potential conflict of interest.
