# Supplementary material for: Synthesis of Oxazinanones: Intramolecular Cyclization of Amino Acid-Derived Diazoketones via Silica-Supported HClO4 Catalysis
Source: Front Chem. 2019 Feb 8;7:62. doi: 10.3389/fchem.2019.00062 (PMC6376066; doi:10.3389/fchem.2019.00062)
Supplement: Supplementary file 1 [file Table_1.DOCX]

Supplementary Material

Synthesis of Oxazinanones: Intramolecular Cyclization of Amino Acid-Derived Diazoketones via Silica-Supported HClO_4_ Catalysis

Rafael D. C. Gallo, Orlando C. Campovilla Jr., Anees Ahmad, Antonio C. B. Burtoloso^*^

*** Correspondence:**antonio@iqsc.usp.br

**SUMMARY**

| **GENERAL INFORMATION** | 4 |
| --- | --- |
| **EXPERIMENTAL PROCEDURE** | 4 |
| **REFERENCES** | 12 |
| **SPECTRA DATA** | 14 |
| **^1^H NMR** (500 MHz, CDCl_3_) Benzyl (*S*)-(4-diazo-3-oxo-1-phenylbutan-2-yl)carbamate **(1)** | 14 |
| **^1^H NMR** (500 MHz, CDCl_3_) Benzyl (*S*)-(3-diazo-2-oxo-1-phenylpropyl)carbamate **(3)** | 15 |
| **^1^H NMR** (500 MHz, CDCl_3_) Benzyl (*S*)-(1-diazo-5-methyl-2-oxohexan-3-yl)carbamate **(4)** | 16 |
| **^1^H NMR** (500 MHz, CDCl_3_) Benzyl (*S*)-(4-diazo-3-oxobutan-2-yl)carbamate **(5)** | 17 |
| **^1^H NMR** (500 MHz, CDCl_3_) Benzyl (*S*)-(1-diazo-4-methyl-2-oxopentan-3-yl)carbamate **(6)** | 18 |
| **^1^H NMR** (500 MHz, CDCl_3_) Benzyl 3-diazo-2-oxopropylcarbamate **(7)** | 19 |
| **^1^H NMR** (500 MHz, CDCl_3_) Methyl (*S*)-4-(((benzyloxy)carbonyl)amino)-6-diazo-5-oxohexanoate **(8)** | 20 |
| **^1^H NMR** (500 MHz, CDCl_3_) Benzyl (*S*)-2-(2-diazoacetyl)pyrrolidine-1-carboxylate **(9)** | 21 |
| **^1^H NMR** (500 MHz, CDCl_3_) Benzyl (*S*)-2-(2-diazoacetyl)piperidine-1-carboxylate **(10)** | 22 |
| **^1^H NMR** (500 MHz, CDCl_3_) Dibenzyl (7-diazo-6-oxoheptane-1,5-diyl)(S)-dicarbamate **(11)** | 23 |
| **^1^H NMR** (500 MHz, CDCl_3_) (*S*)-4-benzyl-1,3-oxazinane-2,5-dione **(2)** | 24 |
| **^13^C NMR** (125 MHz, CDCl_3_) (*S*)-4-benzyl-1,3-oxazinane-2,5-dione **(2)** | 25 |
| **^1^H NMR** (500 MHz, CDCl_3_) (*S*)-4-phenyl-1,3-oxazinane-2,5-dione **(12)** | 26 |
| **^13^C NMR** (125 MHz, CDCl_3_) (*S*)-4-phenyl-1,3-oxazinane-2,5-dione **(12)** | 27 |
| **^1^H NMR** (500 MHz, CDCl_3_) (*S*)-4-isobutyl-1,3-oxazinane-2,5-dione **(13)** | 28 |
| **^13^C NMR** (125 MHz, CDCl_3_) (*S*)-4-isobutyl-1,3-oxazinane-2,5-dione **(13)** | 29 |
| **^1^H NMR** (500 MHz, CDCl_3_) (*S*)-4-methyl-1,3-oxazinane-2,5-dione **(14)** | 30 |
| **^13^C NMR** (125 MHz, CDCl_3_) (*S*)-4-methyl-1,3-oxazinane-2,5-dione **(14)** | 31 |
| **^1^H NMR** (500 MHz, CDCl_3_) (*S*)-4-isopropyl-1,3-oxazinane-2,5-dione **(15)** | 32 |
| **^13^C NMR** (125 MHz, CDCl_3_) (*S*)-4-isopropyl-1,3-oxazinane-2,5-dione **(15)** | 33 |
| **^1^H NMR** (500 MHz, CDCl_3_) (*S*)-tetrahydro-1H-pyrrolo[1,2-c][1,3]oxazine-1,4(3H)-dione **(18)** | 34 |
| **^13^C NMR** (125 MHz, CDCl_3_) (*S*)-tetrahydro-1H-pyrrolo[1,2-c][1,3]oxazine-1,4(3H)-dione **(18)** | 35 |
| **^1^H NMR** (500 MHz, CDCl_3_) (*S*)-tetrahydro-1H,3H-pyrido[1,2-c][1,3]oxazine-1,4(4aH)-dione **(19)** | 36 |
| **^13^C NMR** (125 MHz, CDCl_3_) (*S*)-tetrahydro-1H,3H-pyrido[1,2-c][1,3]oxazine-1,4(4aH)-dione **(19)** | 37 |

# GENERAL INFORMATION

All solvents were dried and distilled prior to use by standard procedures. Reagents were purchased at the highest commercial quality and used without further purification, unless otherwise stated, the compound 19 ethyl diazoacetate was obtained commercially. Reactions were monitored by thin layer chromatography (TLC), carried out on 0.25 mm silica gel plates using UV light as visualizing agent and potassium permanganate in aqueous KOH for staining. Column chromatography was performed using silica gel 60 (particle size 0.063-0.210 mm). Unless stated otherwise, all the yields refer to isolated products after flash column chromatography on silica gel. The solvent mixtures employed in TLC analysis and in flash column chromatography on silica gel purifications are reported as volume by volume and in percentages. Proton nuclear magnetic resonance (^1^H NMR) spectra were recorded using 500 MHz equipment. For ^1^H NMR spectra, chemical shifts (δ) are referenced from TMS (0.00 ppm). Coupling constants (*J*) are reported in Hz. For multiplicities the following abbreviations were used: s, singlet; d, doublet; t, triplet; q, quartet; m, multiplet; dd, double doublet; bs, broad singlet; dt, double triplet. Carbon nuclear magnetic resonance (^13^C NMR) spectra were recorded using a NMR spectrometer at 125 MHz. For ^13^C NMR spectra, chemical shifts (δ) are given from CDCl_3_ (77.0 ppm). Infrared spectra were obtained using FT-IR at 4.0 cm^-1^ resolution and are reported in wavenumbers. Melting points were determined using a digital melting point apparatus and were not corrected. High resolution mass spectra (HRMS) were recorded using electron spray ionization (ESI) (Hybrid linear ion trap–orbitrap FT-MS and QqTOF/MS).

# Experimental Procedure

General procedure for protection of amino acids with [Benzyl chloroformate](https://www.sigmaaldrich.com/catalog/product/aldrich/119938?lang=en&region=US)

6 mmol of the amino acid (1.0 equiv) was transferred to a 125 mL flask. Saturated aqueous NaHCO_3_ (40 mL) was added and stirred for 5 minutes. After that, 14 mmol of benzyl chloroformate (2.3 equiv., 2 mL) was added slowly. The reaction mixture was stirred overnight. After this period, the reaction mixture was washed with Et_2_O (6 x 10 mL), acidified with HCl (36-38%) to pH 1 and extracted with AcOEt (3 x 30 mL). To the AcOEt organic phase was added anhydrous sodium sulfate, the mixture filtered, and the solvent evaporated in a rotary evaporator. The resulting residue was used directly without further purification for the synthesis of the respective diazoketones.

General procedure for the synthesis of diazoketones

In a 50 mL round bottom flask, 2.53 mmol of the protected *N*-Cbz amino acid (1 equiv.) were dissolved in a solution containing 5 mL of dry Et_2_O and 5 mL of dry THF. The system was cooled to -20 °C and maintained under inert argon atmosphere. Then 0.26 g of triethylamine (2.53 mmol, 1.0 equiv., 0.36 mL) and 0.35 g of isobutyl chloroformate (2.53 mmol, 1 equiv., 0.33 mL) as added. The mixture was stirred for 30 minutes and then 13 mL of a 0.4 M ethereal diazomethane solution (5.06 mmol, 2 equiv.) was added slowly. The reaction temperature was raised to room temperature and the mixture was stirred for approximately 3h and was monitored by TLC. The total consumption/evaporation of the diazomethane was verified through the argon purge using a gas bubbler. Finally, the solvent was evaporated and the residue was diluted with diethyl ether, washed with water, treated with saturated NaHCO_3_ (10 mL) and brine (10 mL). The organic phase was dried, evaporated and the corresponding diazoketone was purified by flash column chromatography on silica gel.

General procedure for the synthesis of oxazinanones from diazoketones

Amino acid-derived diazoketone (0.1 mmol, 1 equiv.) was dissolved in HPLC grade MeOH (0.5 mL) in a 5 mL reaction vial. After complete solubilization, 0.31 g (30 mol%) of the catalyst HClO_4_-SiO_2_ (0.14 mmol HClO_4_-SiO_2_) was added. The reaction was stirred at 25 ^0^C for 1 h and was monitored by TLC. The crude reaction mixture was directly purified by flash column chromatography on silica gel (eluent: 8:2 Hex/AcOEt - 100% AcOEt) without any workup.

Preparation of perchloric acid adsorbed on silica gel (HClO_4_– SiO_2_) (Gallo and Burtoloso, 2018)

HClO_4_–SiO_2_ preparation was carried out following the reported procedure. To a suspension of silica gel (10 g, 230–400 mesh) in EtOAc (20 mL) was added HClO_4_ (0.14 g, 1.40 mmol, that correspond to 0.2 g of a 70% aq. solution of HClO_4_) and the mixture was stirred for 30 min at rt. EtOAc was removed under reduced pressure (rotary evaporator) and the residue was heated at 100 °C for 4 h under vacuum to afford HClO_4_–SiO_2_ (0.14 mmol.g^−1^).

#### Benzyl (S)-(4-diazo-3-oxo-1-phenylbutan-2-yl)carbamate (1) (Zheng and Xu, 2014)

**Yield:** 84%. Pale yellow solid. **M.P:** 80–82 °C. **TLC:** Rf = 0.36 (40% EtOAc/hexanes). **^1^H NMR** (500 MHz, CDCl_3_): δ 7.44 – 7.19 (m, 8H), 7.17 (d, *J* = 6.9 Hz, 2H), 5.39 (d, *J* = 6.2 Hz, 1H), 5.21 (s, 1H), 5.08 (s, 2H), 4.48 (d, *J* = 6.3 Hz, 1H), 3.04 (d, *J* = 6.7 Hz, 2H).

#### Benzyl (*S*)-(3-diazo-2-oxo-1-phenylpropyl)carbamate (3) (Crombie et al., 1993)

**Yield:** 85%. Yellow solid. **M.P:** 80–82 °C. **TLC:** Rf = 0.41 (40% EtOAc/hexanes). **^1^H NMR** (500 MHz, CDCl_3_): δ 7.41 – 7.26 (m, 10H), 6.29 (s, 1H), 5.29 (s, 2H), 5.14 – 5.09 (m, 1H), 5.06 – 5.02 (m, 1H).

#### Benzyl (*S*)-(1-diazo-5-methyl-2-oxohexan-3-yl)carbamate (4) (Zheng and Xu, 2014)

**Yield:** 90%. Yellow solid. **M.P:** 66–67 °C. **TLC:** Rf = 0.36 (40% EtOAc/hexanes). **^1^H NMR** (500 MHz, CDCl_3_): δ 7.38 – 7.30 (m, 5H), 5.43 (s, 1H), 5.25 (s, 1H), 5.10 (s, 2H), 4.26 (s, 1H), 1.70 (dq, *J* = 13.1, 6.6 Hz, 1H), 1.58 (ddd, *J* = 13.4, 8.2, 5.1 Hz, 1H), 1.50 – 1.43 (m, 1H), 0.94 (t, *J* = 5.6 Hz, 6H).

#### Benzyl (*S*)-(4-diazo-3-oxobutan-2-yl)carbamate (5) (Zheng and Xu, 2014)

**Yield:** 92%. Yellow solid. **M.P:** 91–92 °C. **TLC:** Rf = 0,30 (40% EtOAc/hexanes). **^1^H NMR** (500 MHz, CDCl_3_): δ 7.37 – 7.31 (m, 5H), 5.48 (s, 1H), 5.42 (s, 1H), 5.10 (d, *J* = 3.0 Hz, 2H), 4.29 (s, 1H), 1.35 (d, *J* = 7.1 Hz, 3H).

**Benzyl (*S*)-(1-diazo-4-methyl-2-oxopentan-3-yl)carbamate** **(6)** (Zheng and Xu, 2014)

**Yield:** 84%. Yellow solid. **M.P:** 72–73 °C. **TLC:** Rf = 0.296 (40% EtOAc/hexanes). **^1^H NMR** (500 MHz, CDCl_3_): δ 7.37 – 7.30 (m, 5H), 5.28 (d, *J* = 9.0 Hz, 1H), 5.16 – 5.12 (m, 2H), 4.35 (dd, *J* = 9.0, 4.5 Hz, 1H), 2.28 – 2.19 (m, 1H), 1.01 (d, *J* = 6.8 Hz, 3H), 0.93 (d, *J* = 6.8 Hz, 3H).

#### Benzyl 3-diazo-2-oxopropylcarbamate (7) (Dola et al., 2017)

**Yield:** 80%. Yellow solid. **M.P:** 66–67 °C. **TLC:** Rf = 0.40 (40% EtOAc/hexanes). **^1^H NMR** (500 MHz, CDCl_3_): δ 7.36 – 7.30 (m, 5H), 5.57 (s, 1H), 5.38 (s, 1H), 5.12 (s, 2H), 3.96 (s, 2H).

#### Methyl (*S*)-4-(((benzyloxy)carbonyl)amino)-6-diazo-5-oxohexanoate (8) (Carra et al., 2008)

**Yield:** 72%. Yellow solid. **M.P:** 105–107 °C. **TLC:** Rf = 0.26 (40% EtOAc/hexanes). **^1^H NMR** (500 MHz, CDCl_3_): δ 7.37 – 7.31 (m, 5H), 5.65 (d, *J* = 7.8 Hz, 1H), 5.51 (s, 1H), 5.10 (s, 2H), 4.31 (s, 1H), 3.66 (s, 3H), 2.46 (ddd, *J* = 12.1, 7.5, 3.8 Hz, 1H), 2.38 (dt, *J* = 16.9, 6.8 Hz, 1H), 2.16 (dtd, *J* = 12.5, 7.7, 5.1 Hz, 1H), 1.85 (dt, *J* = 14.8, 7.1 Hz, 1H).

#### Benzyl (S)-2-(2-diazoacetyl)pyrrolidine-1-carboxylate (9) (Fournier et al., 2010)

**Yield:** 73%. Yellow solid. **M.P:** 58–60 °C. **TLC:** Rf = 0.26 (40% EtOAc/hexanes). **^1^H NMR** (500 MHz, CDCl_3_): mixture of two rotamers in 55:44 ratio: δ 7.41 – 7.27 (m, 5H), 5.48 (s, 0.5H), 5.25 (s, 0.5H), 5.21 – 5.06 (m, 2H), 4.35 (br s, 0.5H), 4.29 (br s, 0.5H), 3.59 – 3.46 (m, 2H), 2.25 – 1.88 (m, 4H).

#### Benzyl (*S*)-2-(2-diazoacetyl)piperidine-1-carboxylate (10) (Ronkin et al., 2010)

**Yield:** 89%. Yellow solid. **M.P:** 72–74 °C. **TLC:** Rf = 0.36 (10% EtOAc/hexanes). **^1^H NMR** (500 MHz, CDCl_3_): mixture of two rotamers in 55:44 ratio: δ 7.40 – 7.29 (m, 5H), 5.41 (s, 0.5H), 5.34 (s, 0.5H), 5.17 (s, 2H), 4.85 (s, 0.5H), 4.74 (s, 0.5H), 4.13 (m, 1H), 3.00 – 2.82 (m, 1H), 2.27 (d, *J* = 12.6 Hz, 1H), 1.69 – 1.33 (m, 6H).

**Dibenzyl (7-diazo-6-oxoheptane-1,5-diyl)(S)-dicarbamate (11)** (Zheng and Xu, 2014)

**Yield:** 65%. Yellow solid. **M.P:** 83–84 °C. **TLC:** Rf = 0.23 (40% EtOAc/hexanes). **^1^H NMR** (500 MHz, CDCl_3_): δ 7.40 – 7.28 (m, 10H), 5.63 (d, *J* = 7.0 Hz, 1H), 5.44 (s, 1H), 5.13 – 5.03 (m, 4H), 4.89 (s, 1H), 4.22 (s, 1H), 3.24 – 3.11 (m, 2H), 1.91 – 1.68 (m, 2H), 1.66 – 1.33 (m, 6H).

#### (*S*)-4-benzyl-1,3-oxazinane-2,5-dione (2)

**Yield:** 83%. Yellow solid. M.P: 118-121 °C. **TLC:** Rf = 0.30 (100% EtOAc). **^1^H NMR** (500 MHz, CDCl_3_): δ 7.37 – 7.28 (m, 3H), 7.21 – 7.17 (m, 2H), 5.74 (s, 1H), 4.61 (dd, *J* = 17.2, 0.5 Hz, 1H), 4.47 (dd, *J* = 17.2, 0.5 Hz, 1H), 4.10 (ddd, *J* = 9.5, 3.9, 1.7 Hz, 1H), 3.25 (dd, *J* = 14.0, 3.9 Hz, 1H), 2.90 (dd, *J* = 14.0, 9.5 Hz, 1H). **^13^C NMR** (125 MHz, CDCl_3_): δ 201.9, 154.2, 134.6, 129.4, 127.9, 71.7, 60.6, 38.2. **IR** ν_max_ (cm^-1^): 3274, 2954, 2923, 2868, 2853, 1714, 1454, 1436, 1391, 1378, 1284, 1244, 1118, 1058, 755, 733, 700. **HRMS** (ESI-TOF) calculated for C_11_H_12_NO_3_ ([M+H]^+^): 206.0817, found: 206.0813.

#### (S)-4-phenyl-1,3-oxazinane-2,5-dione (12)

**Yield:** 84%. Colourless Solid. **M.P:** 122–124 °C. **TLC:** Rf = 0.28 (100% EtOAc). **^1^H NMR** (500 MHz, CDCl_3_): δ 7.46 – 7.35 (m, 5H), 6.17 (s, 1H), 4.98 (d, *J* = 2.1 Hz, 1H), 4.74 (dd, *J* = 17.4, 0.4 Hz, 1H), 4.68 (dd, *J* = 17.4, 0.5 Hz, 1H). **^13^C NMR** (125 MHz, CDCl_3_): δ 199.4, 154.5, 134.5, 129.5, 129.4, 126.7, 71.3, 63.6. **IR** ν_max_ (cm^-1^): 3254, 2854, 2723, 1716, 1464, 1446, 1341, 1338, 1274, 1110, 1008, 743, 701.

#### (*S*)-4-isobutyl-1,3-oxazinane-2,5-dione (13)

**Yield:** 78%. Colourless Solid. **M.P:** 132–134 °C. **TLC:** Rf = 0.36 (100% EtOAc). **^1^H NMR** (500 MHz, CDCl_3_): δ 6.28 (s, 1H), 4.66 (d, *J* = 17.4 Hz, 1H), 4.61 (d, *J* = 17.4 Hz, 1H), 3.90 (ddd, *J* = 9.2, 4.8, 1.9 Hz, 1H), 1.87 – 1.76 (m, 1H), 1.74 – 1.67 (m, 1H), 1.66 – 1.58 (m, 1H), 0.98 (d, *J* = 6.5 Hz, 3H), 0.95 (d, *J* = 6.5 Hz, 3H). **^13^C NMR** (125 MHz, CDCl_3_): δ 203.1, 154.9, 71.7, 57.9, 40.8, 24.2, 23.1, 21.4. **IR** ν_max_ (cm^-1^): 3250, 3170, 2984, 1708, 1439, 1375, 1319, 1105, 1058, 857, 823, 788, 750, 610.

#### (*S*)-4-methyl-1,3-oxazinane-2,5-dione (14)

**Yield:** 66%. Colourless solid. M.P: 68-70 °C. **TLC:** Rf = 0.30 (100% EtOAc). **^1^H NMR** (500 MHz, CDCl_3_): δ 6.24 (s, 1H), 4.66 (d, *J* = 17.5 Hz, 1H), 4.63 (d, *J* = 17.5 Hz, 1H), 4.00 (qd, *J* = 7.0, 1.2 Hz, 1H), 1.44 (d, *J* = 7.0 Hz, 3H). **^13^C NMR** (125 MHz, CDCl_3_): δ 203.0, 154.7, 71.6, 55.2, 17.1. **IR** ν_max_ (cm^-1^): 3264, 3145, 2964, 2926, 1706, 1440, 1395, 1347, 1309, 1145, 1091, 1048, 897, 843, 778, 740, 637.

#### (*S*)-4-isopropyl-1,3-oxazinane-2,5-dione (15)

**Yield:** 70%. Colourless solid. M.P: 47-48 °C. **TLC:** Rf = 0.21 (1:1 Hex/AcOEt). **^1^H NMR** (500 MHz, CDCl_3_): δ 6.73 (s, 1H), 4.65 (d, *J* = 17.2 Hz, 1H), 4.52 (d, *J* = 17.2 Hz, 1H), 3.72 (dd, *J* = 4.2, 2.2 Hz, 1H), 2.33 (heptd, *J* = 6.9, 4.3 Hz, 1H), 1.07 (d, *J* = 7.0 Hz, 3H), 0.99 (d, *J* = 6.8 Hz, 3H). **^13^C NMR** (125 MHz, CDCl_3_): δ 202.5, 155.4, 72.1, 64.9, 32.2, 18.7, 17.0. **IR** ν_max_ (cm^-1^): 3264, 3145, 2964, 2926, 1706, 1440, 1395, 1347, 1309, 1145, 1091, 1048, 897, 843, 778, 740, 637. **HRMS** (ESI-TOF) calculated for C_7_H_12_NO_3_ ([M+H]^+^): 158,0817, found: 158,0810.

#### (*S*)-tetrahydro-1H-pyrrolo[1,2-c][1,3]oxazine-1,4(3H)-dione (18)

**Yield:** 86%. Colourless Solid. **M.P:** 100–102 °C. **TLC:** Rf = 0.40 (100% EtOAc). **^1^H NMR** (500 MHz, CDCl_3_): δ 4.63 (d, *J* = 18.2 Hz, 1H), 4.53 (d, *J* = 18.2 Hz, 1H), 4.04 (t, *J* = 8.0 Hz, 1H), 3.64 – 3.57 (m, 2H), 2.30 (ddt, *J* = 13.1, 7.5, 5.8 Hz, 1H), 2.10 (ddd, *J* = 16.9, 12.9, 8.4 Hz, 1H), 2.01 – 1.94 (m, 2H). **^13^C NMR** (125 MHz, CDCl_3_): δ 204.2, 153.1, 72.0, 62.6, 46.3, 26.92, 23.1. **IR** ν_max_ (cm^-1^): 2956, 2926, 2862, 1727, 1669, 1453, 1380, 1277, 1122, 1073, 1000, 749, 698.

#### (*S*)-tetrahydro-1H,3H-pyrido[1,2-c][1,3]oxazine-1,4(4aH)-dione (19)

**Yield:** 90%. Colourless Solid. **M.P:** 114–116 °C. **TLC:** Rf = 0.42 (100% EtOAc). **^1^H NMR** (500 MHz, CDCl_3_): δ 4.60 (d, *J* = 16.0 Hz, 1H), 4.56 (d, *J* = 16.0 Hz, 1H), 4.23 – 4.18 (m, 1H), 3.71 (dd, *J* = 11.7, 3.3 Hz, 1H), 2.77 (td, *J* = 13.0, 2.9 Hz, 1H), 2.20 – 2.13 (m, 1H), 2.02 – 1.95 (m, 1H), 1.79 – 1.73 (m, 1H), 1.66 – 1.40 (m, 4H). **^13^C NMR** (125 MHz, CDCl_3_): δ 201.8, 153.9, 71.0, 62.7, 46.1, 29.0, 24.1, 23.4. **IR** ν_max_ (cm^-1^): 2956, 2926, 2862, 1727, 1669, 1473, 1360, 1287, 1132, 1075, 1020, 798.

# References

Carra, R. J., Epperson, M. T., and Gin, D. Y. (2008). Application of an intramolecular dipolar cycloaddition to an asymmetric synthesis of the fully oxygenated tricyclic core of the stemofoline alkaloids. *Tetrahedron* 64, 3629–3641. doi:10.1016/j.tet.2008.02.008.

Crombie, L., Haigh, D., Jones, R. C. F., and Mat-Zin, A. R. (1993). Synthesis of the alkaloid homaline in (±) and natural (S,S)-(–) forms, using amination and transamidative ring expansion in liquid ammonia. *J. Chem. Soc., Perkin Trans. 1* 30, 2047–2054. doi:10.1039/P19930002047.

Dola, V. R., Soni, A., Agarwal, P., Ahmad, H., Raju, K. S. R., Rashid, M., et al. (2017). Synthesis and evaluation of chirally defined side chain variants of 7-chloro-4-aminoquinoline to overcome drug resistance in malaria chemotherapy. *Antimicrob. Agents Chemother.* 61, 1–26. doi:10.1128/AAC.01152-16.

Fournier, A. M., Brown, R. A., Farnaby, W., Miyatake-Ondozabal, H., and Clayden, J. (2010). Synthesis of (−)-( S , S )-clemastine by Invertive N → C Aryl Migration in a Lithiated Carbamate. *Org. Lett.* 12, 2222–2225. doi:10.1021/ol100627c.

Gallo, R. D. C., and Burtoloso, A. C. B. (2018). Silica-supported HClO 4 promotes catalytic solvent- and metal-free O–H insertion reactions with diazo compounds. *Green Chem.* 20, 4547–4556. doi:10.1039/C8GC02574F.

Ronkin, S. M., Badia, M., Bellon, S., Grillot, A. L., Gross, C. H., Grossman, T. H., et al. (2010). Discovery of pyrazolthiazoles as novel and potent inhibitors of bacterial gyrase. *Bioorganic Med. Chem. Lett.* 20, 2828–2831. doi:10.1016/j.bmcl.2010.03.052.

Zheng, Y., and Xu, J. (2014). Synthesis of enantiopure free and N-benzyloxycarbonyl-protected 3-substituted homotaurines from naturally occurring amino acids. *Tetrahedron* 70, 5197–5206. doi:10.1016/j.tet.2014.05.098.

**SPECTRA DATA**

**^1^H NMR** (500 MHz, CDCl_3_) Benzyl (*S*)-(4-diazo-3-oxo-1-phenylbutan-2-yl)carbamate **1**

**^1^H NMR** (500 MHz, CDCl_3_) Benzyl (*S*)-(3-diazo-2-oxo-1-phenylpropyl)carbamate **3**

**^1^H NMR** (500 MHz, CDCl_3_) Benzyl (*S*)-(1-diazo-5-methyl-2-oxohexan-3-yl)carbamate **4**

**^1^H NMR** (500 MHz, CDCl_3_) Benzyl (*S*)-(4-diazo-3-oxobutan-2-yl)carbamate **5**

**^1^H NMR** (500 MHz, CDCl_3_) Benzyl (*S*)-(1-diazo-4-methyl-2-oxopentan-3-yl)carbamate **6**

**^1^H NMR** (500 MHz, CDCl_3_) Benzyl 3-diazo-2-oxopropylcarbamate **7**

**^1^H NMR** (500 MHz, CDCl_3_) Methyl (S)-4-(((benzyloxy)carbonyl)amino)-6-diazo-5-oxohexanoate **8**

**^1^H NMR** (500 MHz, CDCl_3_) Benzyl (*S*)-2-(2-diazoacetyl)pyrrolidine-1-carboxylate **9**

**^1^H NMR** (500 MHz, CDCl_3_) Benzyl (*S*)-2-(2-diazoacetyl)piperidine-1-carboxylate **10**

**^1^H NMR** (500 MHz, CDCl_3_) Dibenzyl (7-diazo-6-oxoheptane-1,5-diyl)(S)-dicarbamate **11**

**^1^H NMR** (500 MHz, CDCl_3_) (*S*)-4-benzyl-1,3-oxazinane-2,5-dione **2**

**^13^C NMR** (125 MHz, CDCl_3_) (*S*)-4-benzyl-1,3-oxazinane-2,5-dione **2**

**^1^H NMR** (500 MHz, CDCl_3_) (S)-4-phenyl-1,3-oxazinane-2,5-dione **12**

**^13^C NMR** (125 MHz, CDCl_3_) (*S*)-4-phenyl-1,3-oxazinane-2,5-dione **12**

**^1^H NMR** (500 MHz, CDCl_3_) (*S*)-4-isobutyl-1,3-oxazinane-2,5-dione **13**

**^13^C NMR** (125 MHz, CDCl_3_) (*S*)-4-isobutyl-1,3-oxazinane-2,5-dione **13**

**^1^H NMR** (500 MHz, CDCl_3_) (*S*)-4-methyl-1,3-oxazinane-2,5-dione **14**

**^13^C NMR** (125 MHz, CDCl_3_) (*S*)-4-methyl-1,3-oxazinane-2,5-dione **14**

**^1^H NMR** (500 MHz, CDCl_3_) (*S*)-4-isopropyl-1,3-oxazinane-2,5-dione **15**

**^13^C NMR** (125 MHz, CDCl_3_) (*S*)-4-isopropyl-1,3-oxazinane-2,5-dione **15**

**^1^H NMR** (500 MHz, CDCl_3_) (*S*)-tetrahydro-1H-pyrrolo[1,2-c][1,3]oxazine-1,4(3H)-dione **18**

**^13^C NMR** (125 MHz, CDCl_3_) (*S*)-tetrahydro-1H-pyrrolo[1,2-c][1,3]oxazine-1,4(3H)-dione **18**

**^1^H NMR** (500 MHz, CDCl_3_) (*S*)-tetrahydro-1H,3H-pyrido[1,2-c][1,3]oxazine-1,4(4aH)-dione **19**

**^13^C NMR** (125 MHz, CDCl_3_) (*S*)-tetrahydro-1H,3H-pyrido[1,2-c][1,3]oxazine-1,4(4aH)-dione **19**
